# The effect of using a cycling workstation on mouse dexterity

**DOI:** 10.1371/journal.pone.0220896

**Published:** 2019-08-28

**Authors:** Anna Anderson, Oliver Thornton, Rachel Coats, Antonio Capozio, Sarah Astill

**Affiliations:** 1 School of Biomedical Sciences, Faculty of Biological Sciences, University of Leeds, Leeds, England, United Kingdom; 2 Physiotherapy Department, Leeds Teaching Hospitals NHS Trust, Leeds, England, United Kingdom; 3 School of Psychology, Faculty of Medicine and Health, University of Leeds, Leeds, England, United Kingdom; University of Bologna, ITALY

## Abstract

This study investigated the effect of using a cycling workstation on mouse dexterity, including if and how this changed with practice. Thirty-four healthy adults were allocated to a sitting group (n = 17) or cycling group (n = 17). All participants completed standardised computer tasks on 6 occasions: baseline and final—all participants were seated; practice 1 to 4—sitting group participants were seated, cycling group participants pedalled on an under desk cycle. Three computer tasks were employed: (1) Tracking (continuous task)—participants used the mouse pointer to track a dot in a figure of 8 pattern at 3 different speeds without a guide then with a guide (2) Aiming (discrete task)—participants moved the mouse pointer to a dot which repeatedly disappeared then reappeared again in different locations, creating the outline of a pentagram (3) Steering (continuous task)—participants steered the mouse pointer around two different pathways. Accuracy was measured during the Tracking and Steering tasks as the root mean square error and penalised path accuracy respectively. Speed was measured during the Aiming task as the movement time. Data was analysed using frequentist and Bayes Factor analyses. During the continuous tasks (Tracking and Steering), accuracy was impaired among participants using the cycling workstation, both compared to their accuracy when seated and to the accuracy of participants in the sitting group. In contrast, no deficits in speed were noted among participants using the cycling work station during the discrete task (Aiming). No learning effects were observed among either group for any tasks. These findings suggest using a cycling workstation may impair the accuracy but not speed of mouse use, regardless of task practice. Overall this supports the implementation of cycling workstations in typical office settings, but suggests cycling workstations may impair productivity among workers performing high precision mouse tasks.

## Introduction

Insufficient physical activity increases the risk of multiple non-communicable diseases, shortens life-expectancy and presents a major economic burden [1–3]. Prolonged sedentary behaviour is also a risk factor for many non-communicable diseases and mortality independent of physical activity [4, 5]. Dose response relationships between sedentary behaviour and mortality have been identified, with cardiovascular and all-cause mortality risk increasing particularly rapidly above 6–8 hours per day of total sitting [5]. Furthermore, meeting current physical activity recommendations does not fully negate the increased mortality risk associated with sitting for more than 8 hours per day [6]. The declining levels of physical activity and escalating levels of sedentary behaviour which are currently being observed internationally therefore present major public health issues [7].

The increasing prevalence of sedentary occupations is recognised as a major contributor to these issues [8–10]. For example, daily occupation-related energy expenditure in the US is estimated to have reduced by more than 100 calories between 1960 and 2008 [9], while a survey of UK employees from a wide range of employment sectors found the average daily occupational sitting time was 6.5 hours [10]. The ubiquity of occupational sedentary behaviour has made the workplace a key target for promoting physical activity and reducing sedentary time [8, 11]. It has been argued that ‘activity-permissive’ workstations, such as treadmill or cycling workstations, hold particular promise because of their potential to enable workers to become more active without reducing productivity [8, 12, 13]. This approach is supported by evidence suggesting that breaking up prolonged periods of sedentary behaviour with low intensity physical activity may offer health benefits [14].

Cycling workstations warrant specific attention because they can be created easily at relatively low cost through the use of portable pedal machines [15]. Use of cycling workstations has been shown to offer diverse benefits, such as raising energy expenditure [16], improving response speed [17] and increasing positive affect, motivation and morale [18]. Furthermore, providing sedentary employees with access to a portable pedal machine, motivational website and pedometer has been shown to significantly reduce sedentary time and waist circumference [19].

Previous studies suggest that the use of cycling workstations results in minimal or no impact on the speed and accuracy of typing [16, 17, 20–23]. For example, participants were asked to copy a text from a window in the top half of the computer screen to a Word document situated in the bottom half of the screen and the number of errors and time taken to complete the task noted [20, 21]. Both studies noted no significant detrimental impact on either the speed or accuracy of typing even when cycling concurrently. However, other tasks which were used to simulate those undertaken in an office-based environment were affected by use of a cycling workstation. For example, participants rated the quality of a telephone conversation significantly lower when cycling [20]. Other substantial deficits in the accuracy of computer mouse use [20, 21], and small but significant deficits in the speed of mouse use [16, 20, 21] have also been noted. For example, tasks that have predominantly required participants to move a mouse from one location or target to another as quick as possible show that reaction time is comprised during cycling, and participants also make more errors in making these fast, discrete movements of the mouse [20, 21]. This may reflect the higher precision and eye-hand coordination required during mouse use compared to typing [21]. If such deficits in mouse dexterity persist long-term they may have a detrimental impact on workplace productivity, presenting a potential barrier to the widespread implementation of cycling workstations [16].

The reduction in mouse dexterity that occurs during use of cycling workstations may be due to biomechanical and/or cognitive factors [21]. A seated cycling posture provides a relatively stable base; hence biomechanical factors are likely to be less significant for cycling workstations than treadmill workstations [20, 21]. Cognitive factors are likely to have an important impact due to the dual task nature using a mouse whilst cycling [21]. Undertaking a dual task typically impairs performance of one or both tasks, an effect known as dual-task interference [24]. Dual task interference may be reduced or even eliminated through dual task practice [25]. In line with this, it has been suggested that the deficits in mouse dexterity associated with use of cycling workstations may be reduced through task practice [16, 21]; however previous studies investigating the impact of cycling on mouse dexterity have involved a single cycling session only [16, 20, 21]. Investigating this area is of paramount importance in order to inform the implementation of cycling workstations and guide future research.

The aim of this study was therefore to investigate the impact of using a cycling workstation on mouse dexterity during task practice. We chose 3 tasks which simulate common office-based tasks. In our study participants performed a task which involved making discrete aiming movements, where the mouse moved from one location to the other, and unlike other studies where performance was based upon reaction time, we measured movement time. We also examined tasks which required movements that were continuous in nature, involving moving the mouse along a prescribed path, where we noted how accurately this could be complete. Furthermore, in direct contrast to other studies which have focused on single-session trials, this study examined if and how performance changed with practice across multiple sessions, thus enabling participants to become more familiar with the desk cycle. It was hypothesised that cycling would result in an initial deterioration in the speed and accuracy of mouse use, but that these deficits would improve over the multiple sessions of task practice. Sitting on the other hand (our control group) should lead to no such initial deterioration in performance, so we expect an interaction between group and practice session.

## Materials and methods

### Participants

**Fig 1:**
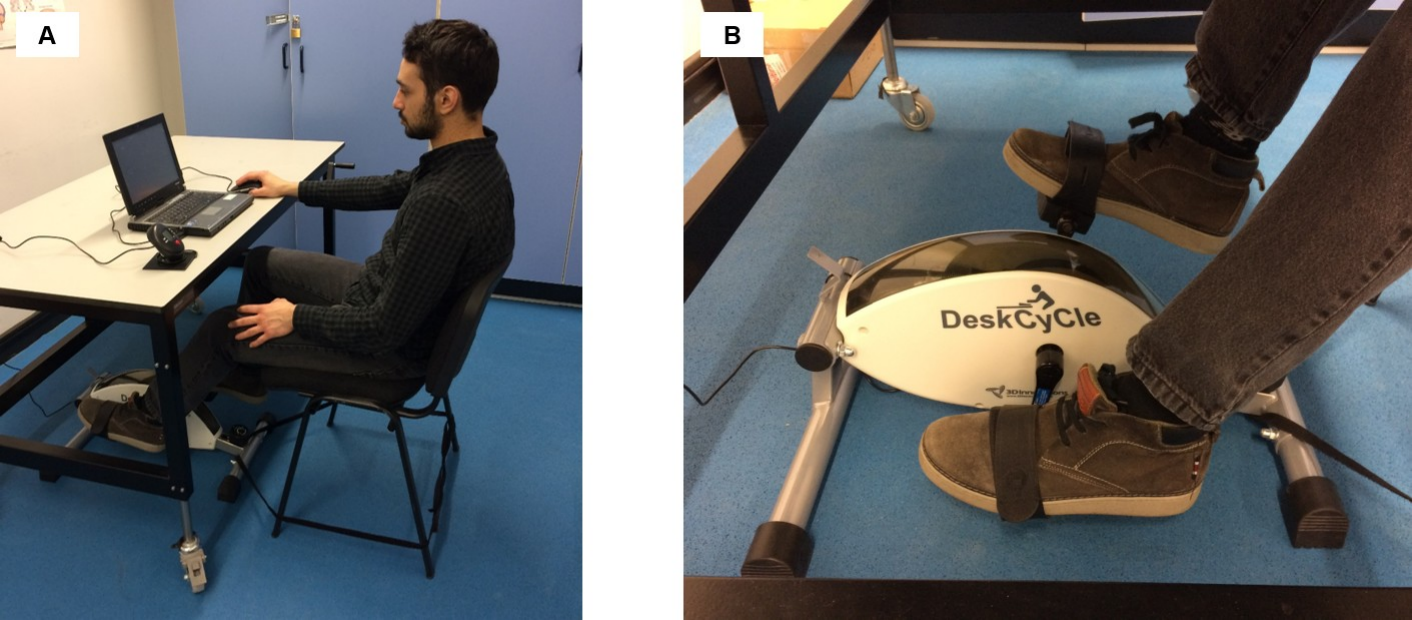
Experimental set up for participants in the cycling group. (A) Overall experimental set up. (B) Close up view of the DeskCycle.

We performed an a priori sample size calculation based on our hypothesis of a difference in performance between the two groups using the software G*Power. The required power was set at 1- β = 0.80 and the level of significance was kept at α = 0.05. Results of the sample size calculation showed that we would need a total sample size of 36 participants to be able to detect a large effect size of 0.4 (Cohen, 1988). Thirty-four participants were recruited via posters advertising the study placed in and around The University of Leeds. Participants were recruited between May 2017 and February 2018. All participants were randomly allocated to either a cycling group (n = 17, females = 7, mean age = 21.72 years, SD = 1.38 years) or a sitting group (n = 17, females = 10, mean age = 22.67 years, SD = 2.91 years). Inclusion criteria included being aged between 18 and 30 years old, in good health at the time of testing and right-handed as assessed by the Edinburgh Handedness Inventory [26]. All participants gave informed consent and the individual in Fig 1 of this manuscript has given written informed consent to publish this image. The study was approved by the University of Leeds Biological Sciences Ethics Committee.

### Apparatus

Motor tasks were performed on a laptop computer (Toshiba Portege, M750, Toshiba, Japan) running the Clinical Kinematic Assessment Tool (CKAT) software [27], a test battery designed to measure tracking, aiming and steering performance. All participants completed the battery by moving a computer mouse, to simulate working at a computer workstation. Participants were seated on a desk chair at a height adjustable desk which was adjusted to ensure leg comfort while completing the tasks i.e. to ensure clearance for the knees while cycling [22]. The laptop computer was placed on the desk in front of the participant at a fixed distance (15 cm) from the table’s edge, with the participants’ wrist resting on the table (See Fig 1A). The participants in the cycling group were on some sessions asked to perform the tasks while pedalling on an under desk cycle (DeskCycle, 3d Innovations, LLC., Colorado, USA) (Fig 1B), the resistance of which was set to 3 (mid-resistance).

### Test battery

The CKAT test battery was completed on every session and lasted approximately 12–15 minutes. The 3 subtests were completed in the following order.

#### 1. Tracking subtest

**Fig 2:**
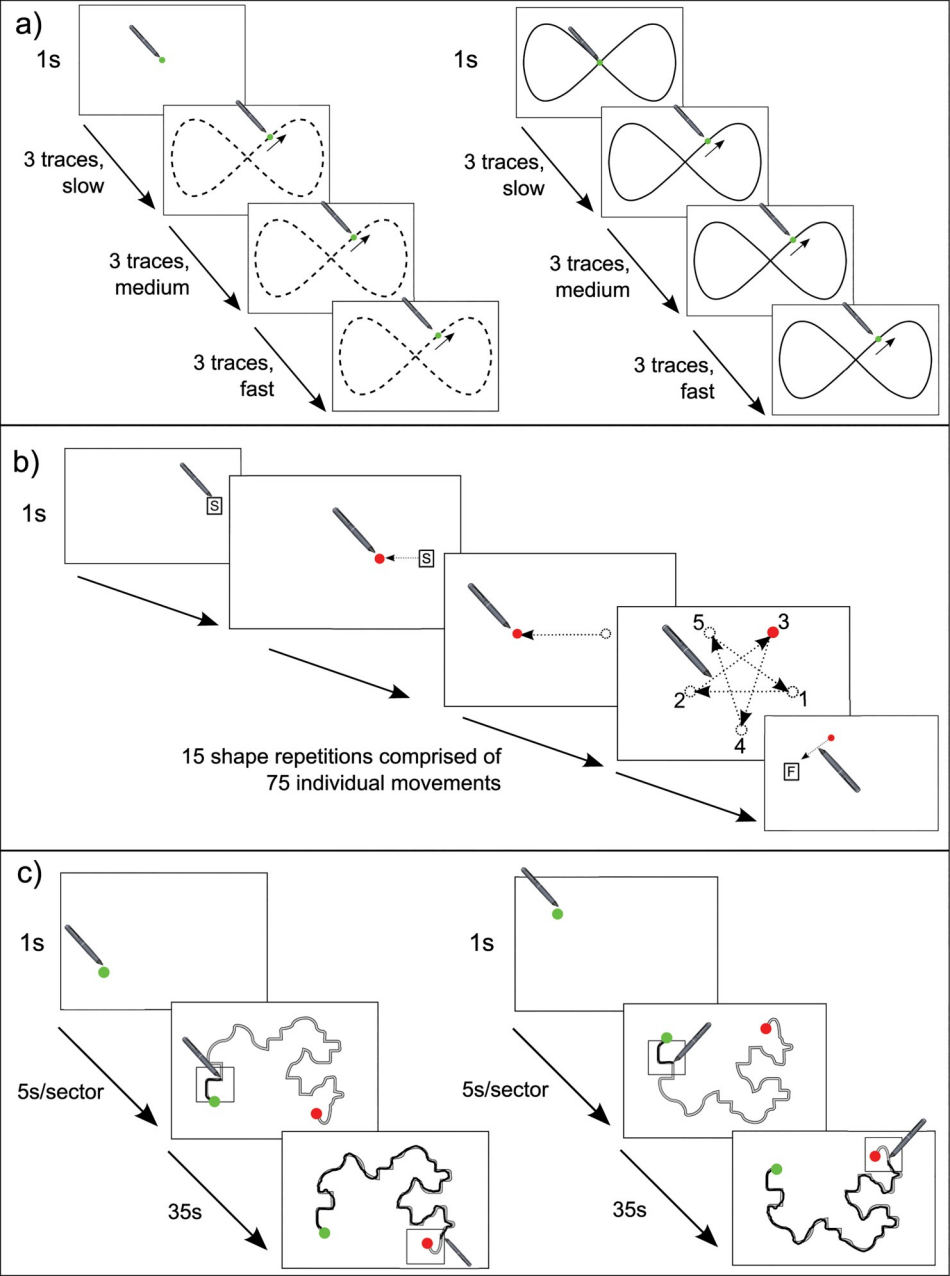
Illustrations of the three subtests included in the Clinical Kinematic Assessment Tool (CKAT) test battery. (A) Tracking (left–no guide; right–with guide); (B) Aiming; (C) Steering (left–shape A; right–shape B). Figure reproduced with permission from Flatters et al. (2014) [28].

Participants were presented with a screen which was completely blank apart from a green dot (diameter = 10mm) at the centre of the screen. They were asked to place the mouse pointer on the dot and, after a 2 second delay, the dot started moving, depicting a figure of 8 pattern (height = 55 mm, width = 110 mm) (Fig 1A). Participants were required to follow the trajectory of the dot while trying to keep the mouse pointer as close to it as possible. Participants were asked to complete this subtest twice, firstly with no guide present (no guide subtest) and secondly with a black guideline depicting the figure of 8 (with guide subtest) (Fig 2).

Each subtest comprised 9 revolutions, including 3 slow speed revolutions (42 mm.s^-1^), 3 medium speed revolutions (84 mm.s^-1)^ and 3 fast speed revolutions (168 mm.s^-1^), always delivered in that order. Accuracy was measured on each trial by calculating the root mean square error (RMSE) as the distance in millimetres between the centre of the green dot and the mouse pointer at each time point, with a smaller RMSE being indicative of better performance. A mean of the 3 trials for each speed was calculated and is presented here.

#### 2. Aiming subtest

At the beginning of this subtest, participants were asked to place the mouse pointer on a dot at the centre of the screen as for the tracking subtest. After a 2 second delay, the dot disappeared and then reappeared again at a different position on the screen. Touching the dot with the mouse pointer resulted in the dot disappearing and then reappearing in a different position again, until 75 aiming movements were completed and the subtest ended. Five consecutive movements created the outline of a pentagram (Fig 2B). The aim of this subtest was to reach the target dots appearing on the screen with the mouse pointer as quickly and accurately as possible. An individual movement time was measured for each movement by calculating the time between the appearance of the dot and the arrival of the mouse pointer at the dot’s position, with a shorter movement time being indicative of better performance. An average movement time was then calculated for each participant as the mean of the participant’s first 50 individual movement times.

#### 3. Steering subtest

At the beginning of this subtest, participants were asked to place the mouse pointer on a dot at the centre of the screen as for the tracking and aiming subtests. After a 1 second delay, a tracing path connecting the green dot (start) to a red dot (finish) appeared. The aim of the subtest was to move the mouse pointer along the path while remaining within the path’s guidelines (4 mm). The mouse pointer produced a black line, providing onscreen feedback to participants about their performance. In addition, participants were asked to pace the speed of their tracing by remaining inside a black box which moved along the path at 5 second intervals throughout the 35 second trial (Fig 2C). The subtest included 6 trials, in which 2 path shapes (A & B) were alternated. Path accuracy (PA) was calculated for each trial as the mean distance in mm across all data points between the traced path and an idealised reference path.

To account for participants who moved the mouse pointer outside the black box (and hence might have completed the trial either too quickly or too slowly), a penalised path accuracy (pPA) for each trial was calculated. The pPA provides a measure of accuracy which takes movement time (MT) into account. The MT was calculated as the time from when the mouse pointer left the start to when it arrived at the finish. The optimal MT, taking the 1 second delay at the start of the trial into account, was 36 seconds. The following formula was therefore used to calculate the pPA:

$$pPA=PA*\left(1+(\frac{abs(MT-36)}{36})\right)$$

The pPA for each shape was then calculated as the mean of the pPA for each of the 3 trials involving that shape, with a lower pPA reflecting better performance.

### Experimental design

All participants completed the CKAT test battery on 6 separate occasions. Consecutive sessions were spaced at least 48 hours apart, with the majority of sessions being spaced between 2 and 5 days apart. At the first session (baseline), all participants were asked to complete the test battery with their dominant hand while sitting with their feet flat on the floor. At the second session (practice 1), participants completed the test battery under different conditions according to their group allocation. Participants in the sitting group were asked to complete the test battery with their dominant hand while sitting with their feet on the floor, as for the baseline session. In contrast, participants in the cycling group were asked to complete the test battery with their dominant hand while pedalling on an under desk cycle.

Prior to using the under desk cycle, participants in the cycling group were asked to lie supine for a 5 minute rest period, at which point their resting heart rate (HR) was measured using a HR monitor (Polar FT2, Polar Electro Oy, Finland). Participants in the cycling group were then asked to complete the CKAT test battery while pedalling at an intensity which raised their HR to 30–40% of their maximum HR reserve using the following formula [29]:

TargetHR=(HRmax−HRrest)×(30−40%)+HRrest

This intensity was chosen because pedalling at lower intensities is more acceptable to office workers than pedalling at higher intensities, due to the increased sweating associated with higher pedalling intensities [23]. HR was continuously monitored by the experimenter. Verbal feedback was provided if a participant’s HR fell outside the specified levels, allowing the participant to increase or decrease their pedalling cadence to bring their HR back into the specified range.

The subsequent 3 sessions (practice 2, practice 3 and practice 4) were identical to practice session 1 in all ways, with participants completing the CKAT test battery while sitting or pedalling depending on their group allocation. A post-practice session (final) was conducted after the 4 practice sessions as per the baseline session.

### Statistical analyses

All frequentist analyses were conducted using IBM SPSS Statistics software (Version 22.0). Normality was assessed using the Kolmogorov Smirnov test for each group separately. Performance on each subtest was analysed using mixed ANOVAs with repeated measures. Group (cycling, sitting) was used as the between subject factor for all subtests, while session (baseline, practice 1, practice 2, practice 3, practice 4, final) was used as a within subjects factor for all subtests. Speed (slow, medium, fast) and guide (no guide, with guide) were included as an additional within subjects factor for the tracking task. Similarly, shape (A, B) was included as an additional within subjects factor for the steering task. The Greenhouse-Geisser correction factor was used when Mauchley’s test indicated a violation of sphericity. Bonferroni corrections were applied to all pairwise comparisons. Significant interactions were explored post-hoc using appropriate inferential statistics (independent t-tests to examine group at each level of session and repeated measures ANOVAs to examine the effect of session on each group separately). When multiple t-tests were performed, the significance level was adjusted by dividing alpha by the number of t-tests performed. Frequentist tests rely on null hypothesis significance testing therefore cannot provide evidence of no effect; only a non-significant result [30]. For this reason, Bayes Factor analyses were conducted alongside frequentist statistics, using JASP (https://jasp-stats.org/). Bayes Factor analyses allow a direct comparison between the null hypothesis and the alternative hypothesis [31, 32]. The results of the frequentist and Bayes Factor analyses are presented together.

## Results

All participants completed all sessions. Path accuracy and movement time data for the first trial of shape A during the steering subtest of practice session 2 was missing for one participant due to a technical failure. This was accounted for in the results by using data from the second and third trials only during calculation of the pPA.

When the sitting group data was assessed using the Kolmogorov Smirnov test, of 54 cells in the analysis design, 14 satisfied the conventional criterion (p < 0.05) indicating deviation from normality (8 out of 36 in the tracking subtest; 0 out of 6 in the aiming subtest; 6 out of 12 in the steering subtest). P-values for the non-normal distributions ranged from 0.000 to 0.040. When the cycling group data was assessed using the Kolmogorov Smirnov test, of 54 cells in the analysis design, (23 out of 36 in the tracking subtest; 0 out of 6 in the aiming subtest; 5 out of 12 in the steering subtest). P-values for the non-normal distributions ranged from 0.000 to 0.043.

### 1. Tracking subtest

There was a significant main effect of speed (F (1, 36) = 434.971; p < 0.001; η_p_^2^ = 0.931, BF_10_ > 10,000). Pairwise comparisons revealed participants were less accurate at the high speed compared to the medium and slow speeds and at the medium speed compared to the slow speed (p < 0.001 for all comparisons). There was a significant main effect of session (F (3, 94) = 2.868; p = 0.042; η_p_^2^ = 0.082, BF_10_ = 2816.0); however pairwise comparisons failed to identify any significant between session differences. There was a significant main effect of group (F (1, 32) = 6.057; p = 0.019; η_p_^2^ = 0.159, BF_10_ = 2.93), with participants in the cycling group being significantly less accurate than participants in the sitting group. There was no significant main effect of guide (F (1, 32) = 0.377, p = 0.544, η_p_^2^ = 0.012, BF_10_ = 0.08). There was a significant session x group interaction (F (3, 94) = 6.213; p = 0.01; η_p_^2^ = 0.163, BF_10_ > 10,000), but no other interactions reached significance (p > 0.05, BF_10_ < 1). The Bayesian analysis indicated strongest support for the model containing session, speed, group, session x group (BF_10_ > 10,000 compared to the null model, and 2.65 times more likely than the next best model containing session, speed, group, session x group, speed x group) (Table 1).

**Table 1:** Tracking. Shows the mean RMSE values for the cycling and sitting groups during the tracking subtests for each speed, with and without the guide, at each session. Smaller RMSE values indicate greater accuracy.

Session	Speed	Mean root mean square error (mean ± SD)			
		Without guide		With guide	
		Sitting group	Cycling group	Sitting group	Cycling group
Baseline	Slow	4.57 ± 0.95	4.65 ± 1.08	4.83 ± 0.73	5.52 ± 2.18
	Medium	6.73 ± 1.43	6.86 ± 2.09	7.22 ± 1.70	7.34 ± 1.75
	Fast	13.36 ± 4.66	12.09 ± 2.47	12.97 ± 2.89	13.23 ± 3.39
Practice 1	Slow	4.41 ± 1.20	6.89 ± 2.66	4.72 ± 1.10	6.90 ± 3.20
	Medium	6.96 ± 2.92	10.40 ± 3.76	6.84 ± 1.79	9.67 ± 4.21
	Fast	11.65 ± 3.65	16.78 ± 6.22	11.99 ± 4.07	15.93 ± 5.95
Practice 2	Slow	4.31 ± 0.91	6.93 ± 3.18	4.61 ± 1.13	6.59 ± 2.36
	Medium	6.34 ± 1.46	8.83 ± 3.04	6.75 ± 1.48	8.40 ± 2.13
	Fast	12.56 ± 5.08	15.39 ± 7.38	11.66 ± 3.28	14.71 ± 2.71
Practice 3	Slow	4.15 ± 0.83	6.77 ± 3.50	4.71 ± 1.08	6.02 ± 2.10
	Medium	6.21 ± 1.18	8.43 ± 2.74	6.74 ± 1.59	7.96 ± 2.06
	Fast	10.81 ± 2.28	13.52 ± 4.68	10.95 ± 2.03	13.67 ± 3.81
Practice 4	Slow	4.07 ± 0.97	6.78 ± 3.18	4.41 ± 1.02	5.49 ± 1.42
	Medium	6.04 ± 1.66	8.89 ± 2.71	6.34 ± 1.68	8.38 ± 2.76
	Fast	11.82 ± 3.53	15.20 ± 6.10	10.70 ± 3.53	14.88 ± 5.04
Final	Slow	4.69 ± 1.63	5.47 ± 3.62	5.45 ± 2.85	4.90 ± 1.66
	Medium	7.22 ± 2.71	7.70 ± 3.65	7.30 ± 2.90	7.44 ± 4.21
	Fast	12.32 ± 4.84	12.67 ± 5.72	11.85 ± 4.16	12.10 ± 4.94

**Fig 3:**
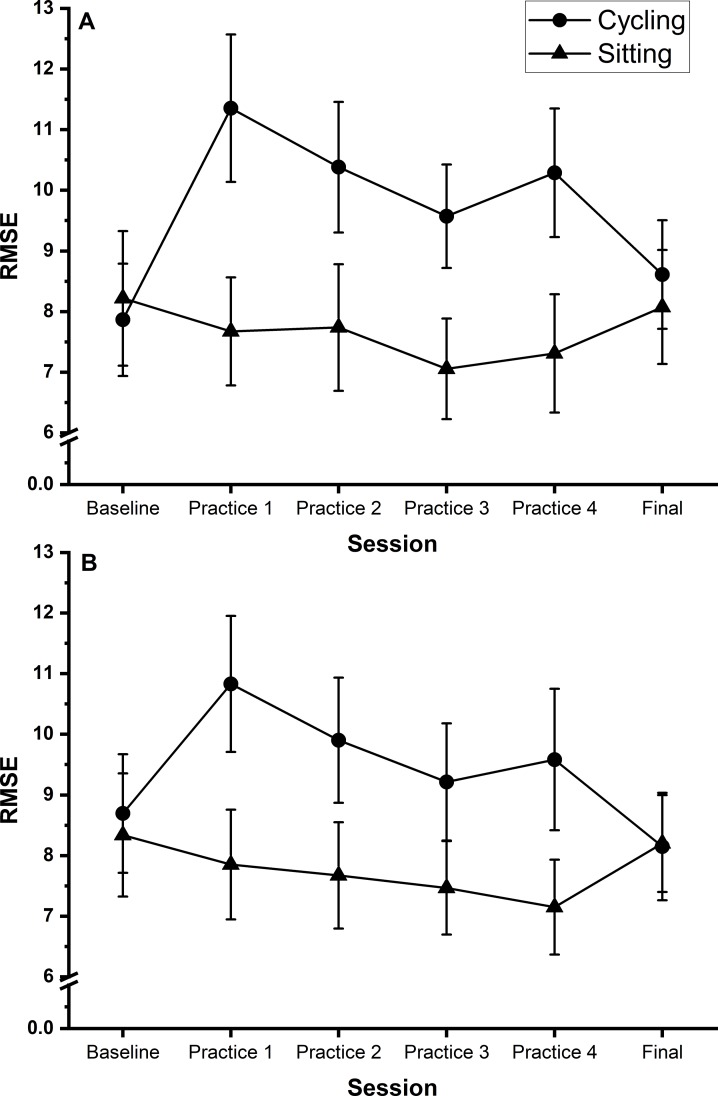
Tracking. Shows line graphs of average root mean square error (RMSE) during the tracking subtest across all sessions. Graph A (top) = no guide; Graph B = with guide. Circles represent the cycling group, triangles the sitting group. Smaller RMSE values indicate greater accuracy. Error bars represent standard error of the mean.

Follow-up independent t-tests to unpack the session x group interaction (collapsed across speed and guide, significance level adjusted to p < 0.0083 due to multiple tests) revealed that participants in the cycling group were significantly less accurate than participants in the sitting group during all the practice sessions (practice 1, p = 0.003, BF_10_ = 13.14; practice 2, p = 0.007, BF_10_ = 6.32; practice 3, p = 0.007, BF_10_ = 7.57; practice 4, p = 0.005, BF_10_ = 9.31), but not the baseline session (p = 0.999, BF_10_ = 0.33) or final session (p = 0.829, BF_10_ = 0.34) (Fig 3).

Repeated measures ANOVAs carried out on each group separately revealed a significant main effect of session for participants in the cycling group (F (2, 40) = 5.092; p = 0.007; η_p_^2^ = 0.241, BF_10_ = 68.70). Pairwise comparisons revealed participants in the cycling group were significantly less accurate during practice session 1 compared to the final session (p = 0.007, BF_10_ = 77.92) and during practice session 4 compared to the final session (p < 0.001, BF_10_ = 592.65). No other comparisons reached significance. There was no significant main effect of session for participants in the sitting group (F (2, 34) = 2.700 p = 0.078; η_p_^2^ = 0.144, BF_10_ = 1.74).

### 2. Aiming subtest

**Fig 4:**
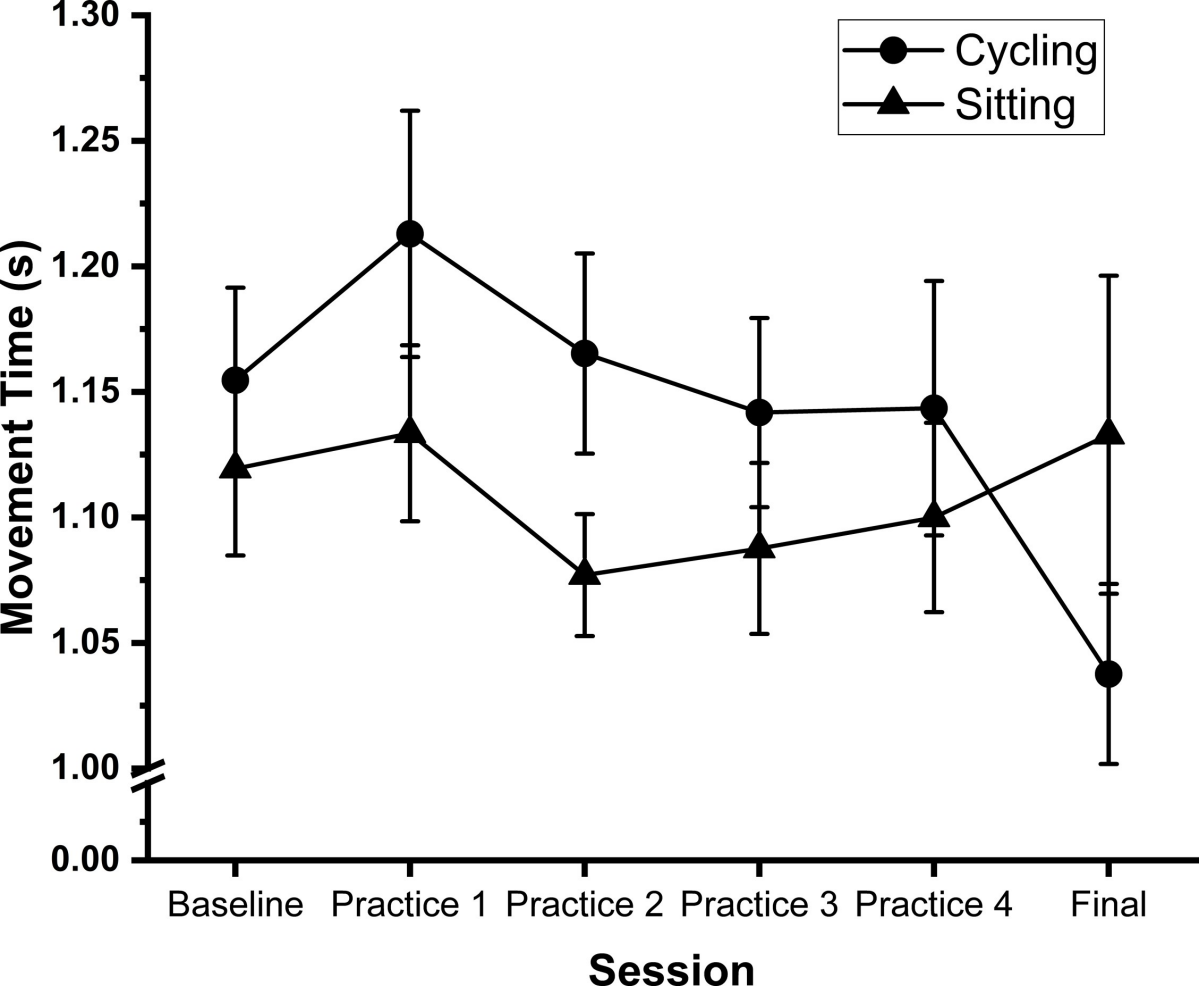
Aiming. Shows line graph of mean movement time during the aiming subtest across all sessions. Circles represent the cycling group, triangles the sitting group. Lower scores correspond to faster (better) performance. Error bars represent standard error of the mean.

There was no significant main effect of session (F (5, 160) = 1.646; p = 0.151; η_p_^2^ = 0.049, BF_10_ = 0.17) or group (F (1, 32) = 0.698; p = 0.410; η_p_^2^ = 0.021, BF_10_ = 0.40). Additionally, there was no significant session x group interaction (F (5, 160) = 2.188; p = 0.058; η_p_^2^ = 0.064, BF_10_ = 0.97) (Fig 4). The Bayesian analysis indicated strongest support for the null model (at least 2.5 times more likely than any other model).

### 3. Steering subtest

There was a significant main effect of group (F (1, 32) = 4.510; p = 0.042; η_p_^2^ = 0.124, BF_10_ = 1.74), with participants in the cycling group being significantly less accurate than participants in the sitting group. No other main effects reached significance (p > 0.05, BF_10_ < 0.65). There was a significant session x group interaction (F (4, 115) = 5.286; p = 0.001; η_p_^2^ = 0.142, BF_10_ > 10,000) but no other interactions reached significance (p > 0.05, BF_10_ < 0.22). The Bayesian analysis indicated strongest support for the model containing, group and session x group (BF_10_ > 10,000) compared to the null model, and 5.01 times more likely than the next best model (group, session x group, shape x group).

**Fig 5:**
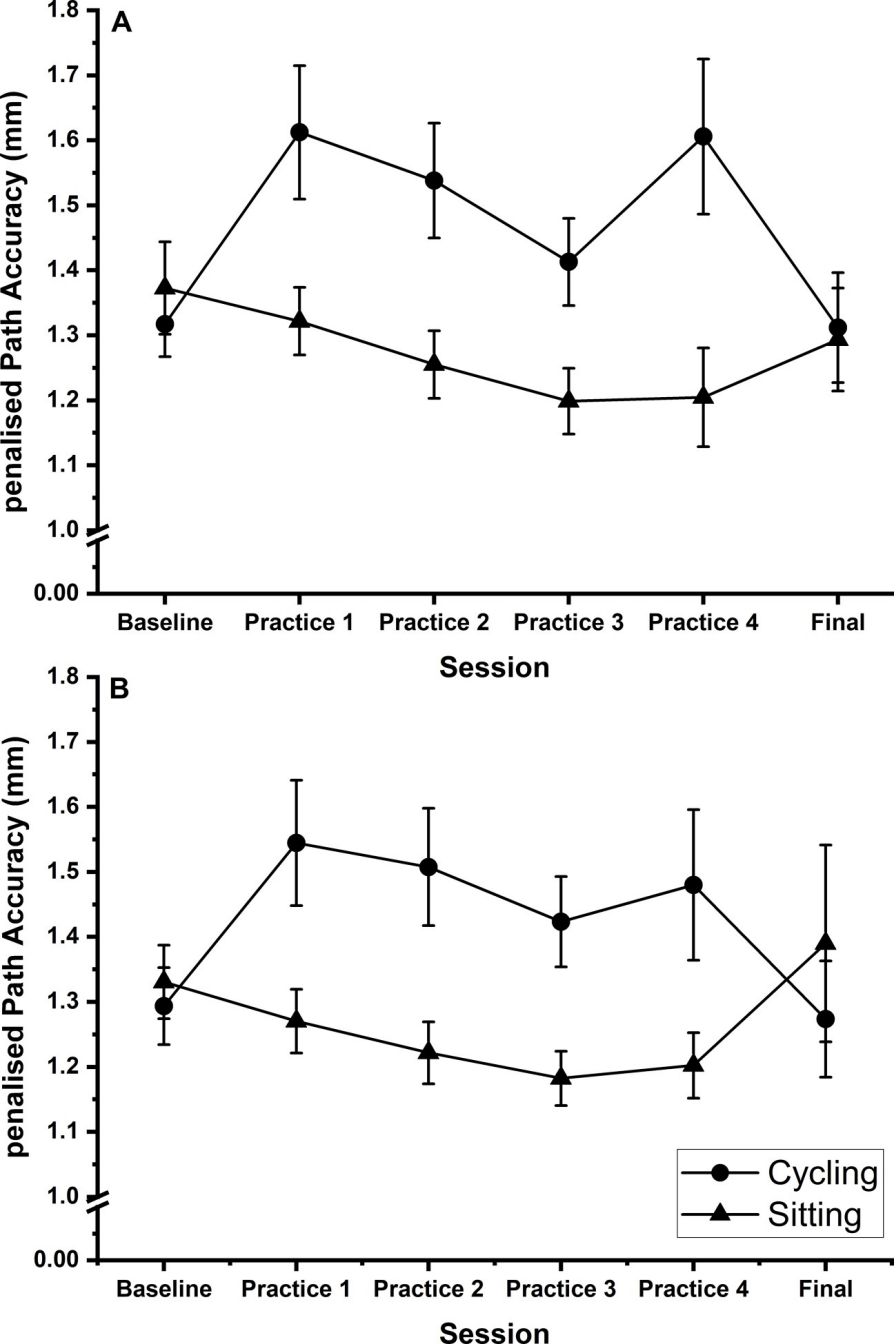
Steering. Shows line graph of mean penalised path accuracy (pPA) during the steering subtest across all sessions. Graph A (top) = shape A data, Graph B (bottom) = shape B. Circles represent the cycling group, triangles the sitting group. Smaller pPA values indicate greater accuracy. Error bars represent standard error of the mean.

Follow-up independent t-tests to unpack the session x group interaction (collapsed across shape, significance level adjusted to p < 0.0083 due to multiple tests) revealed that participants in the cycling group were significantly less accurate than participants in the sitting group for practice sessions 2 (p = 0.007, BF_10_ = 6.44) and 3 (p = 0.007, BF_10_ = 6.60) but not for practice sessions 1 (p = 0.015, BF_10_ = 4.10) and 4 (p = 0.013, BF_10_ = 4.58) although in both cases the Bayes Factors point towards support for the alternative hypothesis over the null, or the baseline session (p = 0.561, BF_10_ = 0.38) or final session (p = 0.722, BF_10_ = 0.35) (Fig 5).

Further repeated measures ANOVAs for each group separately revealed that there was a significant main effect of session for participants in the cycling group (F (5, 80) = 3.746; p = 0.004; η_p_^2^ = 0.190, BF_10_ = 9.25). Pairwise comparisons failed to identify any significant between session differences, but BF_10_ values (>3) show support for their being differences between baseline and practice sessions 1 (BF_10_ = 5.95) and 2 (BF_10_ = 5.78) and between the final session and practice sessions 1 (BF_10_ = 3.37) and 4 (BF_10_ = 3.60). There was no significant main effect of session for participants in the sitting group (F (1.6, 25.2) = 2.969; p = 0.080; η_p_^2^ = 0.157, BF_10_ = 2.66).

## Discussion

The aim of the present study was to investigate the impact of using a cycling workstation on mouse dexterity, and how this changed over multiple sessions of practice. It was hypothesised that cycling would result in an initial deterioration in the speed and accuracy of mouse use, but that these deficits would improve with task practice. This hypothesis was partially supported, as the accuracy of mouse use during continuous tasks (Tracking and Steering subtests) was impaired among participants using the cycling workstation, both in comparison to the same participants’ accuracy when using a standard seated workstation and in comparison to the accuracy of participants in the sitting group. Contrary to the hypothesis however, the speed of mouse use in our discrete computer simulated tasks (Aiming subtest) was not impaired among participants using the cycling workstation, and the deficits in accuracy associated with cycling did not improve with practice.

The finding that using the cycling workstation was associated with deficits in the accuracy of mouse use is consistent with previous studies [20, 21]. Conversely, the lack of impact of cycling on the speed of mouse use contrasts with previous studies [16, 20, 21]. However, the speed deficits associated with cycling in previous studies have all been relatively minor and predominantly relied on reaction time alone. Straker et al. (2009) and Commissaris et al. (2014) assessed both the speed and accuracy of mouse use as participants performed computer tasks at various different workstations in comparison to a standard seated workstation [20, 21]. For the cycling workstations, deficits in the speed of mouse use ranged from 5–8%, whereas deficits in the accuracy of mouse use ranged from 42–68%. Furthermore, Carr et al. (2014) measured the time it took participants to complete three different mouse tasks whilst using a cycling workstation compared to a standard seated workstation, and only identified deficits in the speed of mouse use for one of the mouse tasks [16]. In addition, our Tracking subtest also requires moving at a speed to be able to keep up with the dot so an element of speed might at least partially explain our effects in this condition.

Various factors may have contributed to the lack of speed deficits noted in the current study. In particular, it is possible that the computer tasks used were less challenging than those used in previous studies. This is a likely explanation as the absence of learning effects observed in the current study, even among participants in the sitting group, suggests that participants found the computer tasks relatively easy. In contrast, Straker et al. (2009) reported observing a consistent learning effect when participants completed the computer tasks used in their study six times [21]. However this was based on a small pilot involving only three participants, and minimal details regarding this pilot are available. The type of cycling workstation used in the current study also differs from those used in previous studies, and may have contributed to the lack of speed deficits observed. Participants in the current study were seated on a standard chair whilst using a portable under desk cycle. This may have provided participants with a more stable base than the cycle ergometers and semi-recumbent elliptical trainers used in previous studies [16, 20, 21].

The current study is the first to investigate the impact of using a cycling workstation on mouse dexterity during task practice, over a number of sessions (specifically practice sessions 1–4 in our study). Using a mouse while cycling is a dual task [21]. Dual task practice is a recognised approach for reducing dual task interference and hence improving dual task performance [25]. It was therefore hypothesised that the deficits in mouse dexterity associated with cycling would improve with practice; however this study’s findings do not support this hypothesis. The deficits in the accuracy of mouse use associated with cycling persisted throughout all 4 practice sessions, a finding that was consistent for the no guide and with guide tracking subtests and both shapes used in the steering subtest. The reasons for this lack of improvement with practice were not investigated in the current study. It could be argued that four practice sessions may have been insufficient for the benefits of dual task practice to be achieved. Alternatively, it is possible that biomechanical rather than cognitive factors were primarily responsible for the accuracy deficits observed. The latter explanation is particularly plausible given that some participants had to cycle at a high cadence to maintain the target intensity of 30–40% heart rate reserve. Cycling at a high cadence is likely to have resulted in considerable trunk movement, providing a less stable base for upper limb movements and hence potentially impairing the accuracy of mouse use.

The persistent deficits in the accuracy of mouse use identified in this study could have implications for the implementation of cycling workstations in office settings. However, it is unknown whether the accuracy deficits observed would result in a practically relevant reduction in productivity. The impact of cycling workstations on productivity is likely to vary according to the specific workplace demands, and may only be significant for workers who perform high precision mouse tasks for prolonged periods of time, such as graphic designers. Furthermore, this study’s finding that the speed of mouse use was not impaired by cycling suggests that the productivity of workers performing typical office-based tasks is likely to be minimally affected by cycling. This lends support to the suggestions that cycling workstations could provide a feasible approach for increasing physical activity and reducing sedentary behaviour in typical office settings.

The above findings should however be interpreted with caution as this study presents a number of limitations. One of the limitations of the study is the small sample size, as being underpowered would compromise the reliability of the results [33]. We tested a total number of 34 participants based on sample size calculation aimed at detecting a strong effect of Group in the speed/accuracy of the subtests. Therefore, a possible small/medium effect of Group on the performance in the Aiming subtest would have been undetected due to our total number of participants. However, result from the Bayesian analysis support our conclusion that cycling at a moderate intensity does not significantly affect mouse speed. In addition, the sample consisted of university students only, limiting the generalisability of the findings to office workers. The laboratory set-up of the study also limits its external validity. For example, participants were asked to cycle at a set intensity; however participants in a previous study reported that cycling a set speed was distracting [21]. As highlighted above, the use of 4 practice sessions only is a further limitation of this study. We must also consider the fact that the chair used in the current study did not have arm rests (see Fig 1A), while many office based chairs do. It is generally assumed that chair arms are designed to aid stability of the forearm [34], and in our study this could have reduced variability at the wrist (e.g. decreased RMSE in the tracking task). However, a more contemporary explanation of the dynamic interplay between postural and arm control suggests that the lack of arm rest could result in the participants having a higher level of functional variability in the movements of the shoulder and elbow joints to ensure endpoint accuracy [35, 36]. Future work might wish to ascertain the effect chair arms have on wrist accuracy, however it should be noted that recent research suggests that more dynamic chairs designed to allow workers to more movement when working increase activity levels of the participants with no detrimental effect on performance [34].

Given the significant benefits cycling workstations offer, future research to address these limitations and explore additional benefits/drawbacks of cycling workstations is clearly warranted. In particular, there is a need for studies investigating the impact of cycling workstations on objective measures of work performance to be carried out over longer periods of time, ideally in a diverse range of real workplace settings. The findings of this study suggest biomechanical factors may make a significant contribution to the impaired accuracy of mouse use associated with cycling. It would therefore be of benefit to explore the impact of providing workers with instructions and training in the optimal technique for using cycling workstations.

## Conclusions

Implementation of cycling workstations provides a particularly promising approach for increasing physical activity levels and reducing sedentary behaviour, but may impair how accurately and quickly workers can use a computer mouse. The current study is the first to investigate the impact of using a cycling workstation on mouse dexterity over multiple practice sessions. The results suggest that cycling significantly impairs the accuracy of mouse use, regardless of task practice. This could have a negative impact on workplace productivity, but this may only be significant in settings where workers are required to perform high precision mouse tasks for prolonged periods of time. No significant deficits in the speed of mouse use were observed, even for the first practice session. This suggests that the impact of cycling workstations on productivity is likely to be minimal in typical office settings, supporting the implementation of cycling workstations. These findings should be interpreted cautiously however, as this study presents a number of limitations. In light of these limitations and the considerable benefits cycling workstations offer, future research is warranted to build on this study’s findings and explore additional benefits/drawbacks of cycling workstations.

## Supporting information

S1 Dataset Processed data. This excel file contains the mean for each participant in each session across all tasks. A key can be found in the second sheet.
(XLSX)

## References

[1] Lee IM, Shiroma EJ, Lobelo F, Puska P, Blair SN, Katzmarzyk PT, et al Effect of physical inactivity on major non-communicable diseases worldwide: an analysis of burden of disease and life expectancy. Lancet. 2012;380(9838):219–29. 10.1016/S0140-6736(12)61031-9 22818936 PMC3645500

[2] Arem H, Moore SC, Patel A, Hartge P, Berrington de Gonzalez A, Visvanathan K, et al Leisure time physical activity and mortality: a detailed pooled analysis of the dose-response relationship. JAMA Intern Med. 2015;175(6):959–67. 10.1001/jamainternmed.2015.0533 25844730 PMC4451435

[3] Ding D, Lawson KD, Kolbe-Alexander TL, Finkelstein EA, Katzmarzyk PT, van Mechelen W, et al The economic burden of physical inactivity: a global analysis of major non-communicable diseases. Lancet. 2016;388(10051):1311–24. 10.1016/S0140-6736(16)30383-X 27475266

[4] Biswas A, Oh PI, Faulkner GE, Bajaj RR, Silver MA, Mitchell MS, et al Sedentary time and its association with risk for disease incidence, mortality, and hospitalization in adults: a systematic review and meta-analysis. Ann Intern Med. 2015;162(2):123–32. 10.7326/M14-1651 25599350

[5] Patterson R, McNamara E, Tainio M, de Sá TH, Smith AD, Sharp SJ, et al Sedentary behaviour and risk of all-cause, cardiovascular and cancer mortality, and incident type 2 diabetes: a systematic review and dose response meta-analysis. Eur J Epidemiol. 2018. 10.1007/s10654-018-0380-1 PMC6133005 29589226

[6] Ekelund U, Steene-Johannessen J, Brown WJ, Fagerland MW, Owen N, Powell KE, et al Does physical activity attenuate, or even eliminate, the detrimental association of sitting time with mortality? A harmonised meta-analysis of data from more than 1 million men and women. Lancet. 2016;388(10051):1302–10. 10.1016/S0140-6736(16)30370-1 27475271

[7] Ng SW, Popkin BM. Time use and physical activity: a shift away from movement across the globe. Obes Rev. 2012;13(8):659–80. 10.1111/j.1467-789X.2011.00982.x 22694051 PMC3401184

[8] Keadle SK, Conroy DE, Buman MP, Dunstan DW, Matthews CE. Targeting Reductions in Sitting Time to Increase Physical Activity and Improve Health. Med Sci Sports Exerc. 2017;49(8):1572–82. 10.1249/MSS.0000000000001257 28272267 PMC5511092

[9] Church TS, Thomas DM, Tudor-Locke C, Katzmarzyk PT, Earnest CP, Rodarte RQ, et al Trends over 5 decades in U.S. occupation-related physical activity and their associations with obesity. PLoS One. 2011;6(5):e19657 10.1371/journal.pone.0019657 21647427 PMC3102055

[10] Kazi A, Duncan M, Clemes S, Haslam C. A survey of sitting time among UK employees. Occup Med (Lond). 2014;64(7):497–502. 10.1093/occmed/kqu099 25135938

[11] Buckley JP, Hedge A, Yates T, Copeland RJ, Loosemore M, Hamer M, et al The sedentary office: an expert statement on the growing case for change towards better health and productivity. Br J Sports Med. 2015;49(21):1357–62. 10.1136/bjsports-2015-094618 26034192

[12] Neuhaus M, Eakin EG, Straker L, Owen N, Dunstan DW, Reid N, et al Reducing occupational sedentary time: a systematic review and meta-analysis of evidence on activity-permissive workstations. Obes Rev. 2014;15(10):822–38. 10.1111/obr.12201 25040784

[13] Ojo SO, Bailey DP, Chater AM, Hewson DJ. The Impact of Active Workstations on Workplace Productivity and Performance: A Systematic Review. Int J Environ Res Public Health. 2018;15(3). 10.3390/ijerph15030417 PMC5876962 29495542

[14] Chastin SF, Egerton T, Leask C, Stamatakis E. Meta-analysis of the relationship between breaks in sedentary behavior and cardiometabolic health. Obesity (Silver Spring). 2015;23(9):1800–10. 26308477 10.1002/oby.21180

[15] Carr LJ, Walaska KA, Marcus BH. Feasibility of a portable pedal exercise machine for reducing sedentary time in the workplace. Br J Sports Med. 2012;46(6):430–5. 10.1136/bjsm.2010.079574 21324889

[16] Carr L, Maeda H, Luther B, Rider P, Tucker S, Leonhard C. Acceptability and effects of a seated active workstation during sedentary work: A proof of concept study. 2014;7(1):2–15.

[17] Torbeyns T, de Geus B, Bailey S, De Pauw K, Decroix L, Van Cutsem J, et al Cycling on a Bike Desk Positively Influences Cognitive Performance. PLoS One. 2016;11(11):e0165510 10.1371/journal.pone.0165510 27806079 PMC5091773

[18] Pilcher JJ, Baker VC. Task Performance and Meta-Cognitive Outcomes When Using Activity Workstations and Traditional Desks. Front Psychol. 2016;7:957 10.3389/fpsyg.2016.00957 27445921 PMC4914547

[19] Carr LJ, Karvinen K, Peavler M, Smith R, Cangelosi K. Multicomponent intervention to reduce daily sedentary time: a randomised controlled trial. BMJ Open. 2013;3(10):e003261 10.1136/bmjopen-2013-003261 24141969 PMC3808782

[20] Commissaris DA, Könemann R, Hiemstra-van Mastrigt S, Burford EM, Botter J, Douwes M, et al Effects of a standing and three dynamic workstations on computer task performance and cognitive function tests. Appl Ergon. 2014;45(6):1570–8. 10.1016/j.apergo.2014.05.003 24951234

[21] Straker L, Levine J, Campbell A. The effects of walking and cycling computer workstations on keyboard and mouse performance. Hum Factors. 2009;51(6):831–44. 10.1177/0018720810362079 20415158

[22] Elmer SJ, Martin JC. A cycling workstation to facilitate physical activity in office settings. Appl Ergon. 2014;45(4):1240–6. 10.1016/j.apergo.2014.03.001 24681071

[23] Koren K, Pišot R, Šimunič B. Active workstation allows office workers to work efficiently while sitting and exercising moderately. Appl Ergon. 2016;54:83–9. 10.1016/j.apergo.2015.11.013 26851467

[24] Watanabe K, Funahashi S. Neural mechanisms of dual-task interference and cognitive capacity limitation in the prefrontal cortex. Nat Neurosci. 2014;17(4):601–11. 10.1038/nn.3667 24584049

[25] Strobach T, Torsten S. Mechanisms of Practice-Related Reductions of Dual-Task Interference with Simple Tasks: Data and Theory. Adv Cogn Psychol. 2017;13(1):28–41. 10.5709/acp-0204-7 28439319 PMC5385484

[26] Oldfield RC. The assessment and analysis of handedness: the Edinburgh inventory. Neuropsychologia. 1971;9(1):97–113. 5146491 10.1016/0028-3932(71)90067-4

[27] Culmer PR, Levesley MC, Mon-Williams M, Williams JH. A new tool for assessing human movement: the Kinematic Assessment Tool. J Neurosci Methods. 2009;184(1):184–92. 10.1016/j.jneumeth.2009.07.025 19646475

[28] Flatters I, Hill LJ, Williams JH, Barber SE, Mon-Williams M. Manual control age and sex differences in 4 to 11 year old children. PLoS One. 2014;9(2):e88692 10.1371/journal.pone.0088692 24523931 PMC3921207

[29] American College of Sports Medicine. ACSM's Guidelines for Exercise Testing and Prescription. 8th edition ed. New York: Wolters Kluwer/Lippincott, Williams and Wilkins; 2010.

[30] Barchard KA. Null Hypothesis Significance Testing Does Not Show Equivalence. 2015;15(1):418–21.

[31] Mulder J, Wagenmakers E-J. Editors’ introduction to the special issue “Bayes factors for testing hypotheses in psychological research: Practical relevance and new developments.”. Journal of Mathematical Psychology 2016 p. 1–5.

[32] Wagenmakers EJ, Marsman M, Jamil T, Ly A, Verhagen J, Love J, et al Bayesian inference for psychology. Part I: Theoretical advantages and practical ramifications. Psychon Bull Rev. 2018;25(1):35–57. 10.3758/s13423-017-1343-3 28779455 PMC5862936

[33] Button KS, Ioannidis JP, Mokrysz C, Nosek BA, Flint J, Robinson ES, et al Power failure: why small sample size undermines the reliability of neuroscience. Nat Rev Neurosci. 2013;14(5):365–76. 10.1038/nrn3475 23571845

[34] Grooten WJA, Äng BO, Hagströmer M, Conradsson D, Nero H, Franzén E. Does a dynamic chair increase office workers' movements?—Results from a combined laboratory and field study. Appl Ergon. 2017;60:1–11. 10.1016/j.apergo.2016.10.006 28166867

[35] van Emmerik RE, van Wegen EE. On the functional aspects of variability in postural control. Exerc Sport Sci Rev. 2002;30(4):177–83. 12398115 10.1097/00003677-200210000-00007

[36] Britten L, Shire K, Coats RO, Astill SL. The effect of standing desks on manual control in children and young adults. Gait Posture. 2016;48:42–6. 10.1016/j.gaitpost.2016.04.027 27477706

